# Efficacy of dexmedetomidine versus midazolam when combined with butorphanol for sedation and analgesia during burn dressing changes: A randomized clinical trial

**DOI:** 10.3389/fphar.2022.965441

**Published:** 2022-09-07

**Authors:** Xianchao Ding, Hengfeng Cui, Peng Ma, Xi Chen, Yan Sun, Minye Qu, Zhixin Yan

**Affiliations:** ^1^ Department of Burn and Plastic Surgery, The Affiliated Hospital of Jiangsu University, Zhenjiang, Jiangsu, China; ^2^ Department of General Surgery, Third People’s Hospital of Yancheng, Yancheng, Jiangsu, China; ^3^ Department of Anesthesiology, The Affiliated Hospital of Jiangsu University, Zhenjiang, Jiangsu, China; ^4^ Department of Surgery, The Hospital of Jiangsu University, Zhenjiang, Jiangsu, China; ^5^ Department of Nosocomial Infection Management, The Affiliated Hospital of Jiangsu University, Zhenjiang, Jiangsu, China; ^6^ Department of Traditional Chinese Medicine, The Affiliated Hospital of Jiangsu University, Zhenjiang, Jiangsu, China

**Keywords:** dexmedetomidine, midazolam, burn dressing change, sedation, analgesia

## Abstract

**Objective:** The aim of this study was to compare dexmedetomidine-butorphanol (DB) and midazolam-butorphanol (MB) combinations for sedation, and analgesia in burn patients undergoing dressing changes.

**Methods:** A total of 56 ASA I–II burn patients were included in this single-center randomized clinical trial. The ages of these patients were between 20 and 60 years. TBSA ranged from 10% to 50%. They were randomized to group DB and group MB during dressing change. In the DB group, each patient received a bolus dose of dexmedetomidine (0.5 μg kg^−1^) and intermittent boluses of butorphanol (20 μg kg^−1^). In the MB group, each patient received a bolus dose of midazolam (0.05 mg kg^−1^) and intermittent boluses of butorphanol (20 μg kg^−1^). The primary outcomes were sedation scores and pain scores. The second outcomes were vital signs, side effects, and butorphanol consumption.

**Results:** The sedation scores of these two groups did not differ significantly (*p* > 0.05), and the pain scores of these groups were not significantly different (*p* > 0.05). More patients had hypotension in the DB group than in the MB group (6 versus 0, *p* = 0.01), but the number of patients who had respiratory depression was higher in the MB group compared with the DB group (4 versus 0, *p* = 0.038). Butorphanol consumption in the MB group was higher than in the DB group (*p* = 0.025).

**Conclusion:** Dexmedetomidine is comparable to midazolam when combined with butorphanol in burn patients during dressing change. Compared with midazolam, it has the advantage of opioid-sparing effect.

**Clinical Trial Registration**: [http://www.chictr.org.cn/showproj.aspx&proj=130622], identifier [ChiCTR2100049325].

## Introduction

Dressing change and wound debridement are common practices in patients with burn injury. It can cause severe pain which is a physiological and psychological burden to the patient. This kind of burn pain has been termed procedure pain, it is mainly caused by the stimulus of chemical nociceptors and mechanoreceptors in the burn wound. Lots of inflammatory cytokines such as TNF-a, IL-1b, IL-6, PGE2, and Histamine are involved in the mechanism of burn pain (Morgan et al., 2018). Elevated metabolic level is often followed by burn injury, in which state the concentration of plasma catecholamines, glucagon, and cortisol are increased (Williams and Herndon, 2017). Inadequate pain control may lead to hypermetabolic state and post-traumatic stress disorder, thus, pain and anxiety management is an important issue for burn treatment. Various drugs have been used in burn pain treatment. Which include acetaminophen, opioids, benzodiazepines, propofol, ketamine, dexmedetomidine and Griggs et al. (2017) Opioids are the cornerstone of pain treatment in burn patients, but it has lots of side effects such as nausea and vomiting. Tolerance to opioids may happen when they were used repeatedly and continuously (Wibbenmeyer et al., 2015). The guidelines for burn pain management are different between countries and regions, but the use of anxiolytic drugs in conjunction with opioids is recommended (James and Jowza, 2017).

Midazolam is one of the commonly used sedatives during burn dressing change. The benefit of midazolam lies in its fast onset time for sedation (Conway et al., 2016). But it still has shortcomings. Prolonged sedation would result when it was administered repeatedly. The respiratory depression effect of midazolam is also well known. Thus, the development of new sedatives and regimens is urgently needed.

Dexmedetomidine is a selective α2 adrenergic agonist which has a moderate sedation effect (Liu et al., 2021). The sedation produced by dexmedetomidine is arousable (Khalil et al., 2016). Except for its sedation effect, it also has an analgesic effect. Dexmedetomidine has gained popularity in various kinds of diseases (Su et al., 2016), but its clinical use in burn treatment is still limited. Jiang et al. (2019) reported that it can be successfully used for perioperative sedation in burn patients. Asmussen et al. (2013) found the beneficial effects of dexmedetomidine in burn patients. Retrouvey and Shahrokhi (2015) suggested dexmedetomidine as an adjunct to opioids in the treatment of burn pain.

Herein, we hypothesized that dexmedetomidine in combination with butorphanol might provide better sedation and analgesic effects during burn dressing change than midazolam in combination with butorphanol, and the former would reduce butorphanol consumption. The strengths and shortcomings of this combination drug therapy would get clarification from this trial.

## Materials and methods

### Study population

This clinical trial was approved by the ethics committee of the Affiliated Hospital of Jiangsu University and was registered at the Chinese Clinical Trial Registry (ChiCTR2100049325). It was carried out on inpatients with second-degree to third-degree burns. Following the Declaration of Helsinki, written informed consent was obtained from each patient enrolled in this study. This study was reported following Consolidated Standards of Reporting Trials (CONSORT) guidelines. Inclusion criteria were: burn patients scheduled to undergo dressing change; TBSA ranged from 10% to 50%; ASA physical status I-II; age above 20 years and less than 60 years. Exclusion criteria were: ASA physical status III-IV; less than 20 years old or more than 60 years old; allergy to drugs administered in this trial; sedative drug and analgesic drug abuse; did not cooperate; having severe diseases such as liver or renal insufficiency.

### Study procedure

The dressing change procedures were performed in the ward with intensive care equipment. Patients were allocated to the dexmedetomidine-butorphanol (DB) group or the midazolam-butorphanol (MB) group by the computer-generated random numbers in the ratio of 1:1. The randomized sequence was stored in sealed envelopes. On the day of the dressing change, an envelope was open and drugs were administered by one anesthesiologist according to the allocated group, results were assessed and recorded by another anesthesiologist blinded to the assignment. Patients were not allowed to eat 6 h before and after the procedure. In the DB group, patients received a bolus dose of 0.5 μg kg^−1^ dexmedetomidine intravenously for 10 min, then 20 mg kg^−1^ butorphanol was intravenously injected. In the MB group, patients received a bolus dose of 0.05 mg kg^−1^ midazolam intravenously for 10 min, and then 20 mg kg^−1^ butorphanol was injected. The dressing change procedure began immediately after butorphanol had been injected. During the procedure, we remove the outer dressing and the inner dressing, after cleaning the burn wound with saline, we cover the wound with new dressings. No debridement was performed during the dressing change. Patients were monitored by electrocardiography, noninvasive blood pressure, thoracic impedance, and pulse oximetry. Oxygen is supplied by a nasal catheter, and their heart rate (HR), mean blood pressure (MBP), respiratory rate (RR), and pulse blood oxygen saturation (SpO_2_) were recorded. The Ramsay Sedation Scale (RSS) was used to assess the depth of sedation. The level of sedation ranged from 1 = severe agitation to 6 = deep coma. The Visual Analogue Scale (VAS) was used to assess the degree of pain, it ranged from 0 = no pain to 10 = worst pain. The measurements were recorded before drugs were administered (baseline), at the beginning of the procedure, at 5, 10, 15, and 20 min of the procedure. If VAS was recorded above 5, then 10 μg kg^−1^ butorphanol was intravenously injected as a remedy. Adverse events such as nausea and vomiting, hypotension, bradycardia, dizziness, respiratory depression, and desaturation were recorded. They were recorded throughout the procedure. The systolic blood pressure of less than 90 mmHg was considered to be hypotension and 10 μg min^−1^ norepinephrine was given intravenously as a rescue. The heart rate of fewer than 50 beats per minute was considered to be bradycardia and 0.01 mg kg^−1^ atropine was given intravenously. The SpO_2_ of less than 90% was considered to be desaturation and a nasal cannula was changed to an oxygen mask. The primary outcomes were RSS and VAS. The second outcomes were side effects and butorphanol consumption. For every enrolled patient, a dressing change was performed once every 2 days; five consecutive dressing changes were recorded. The data recorded in five consecutive procedures were used for analyses.

### Sample size calculation

A pilot study was conducted to determine the sample size. A standard deviation of one was recorded for the difference in RSS. At the two-sided significant level of 0.05, and the power of 90%, at least 23 cases in each group were required. In the hypothesis of the 20% dropout rate, 30 cases in each group were needed.

### Statistical analysis

Data were presented as numbers for categorical variables, and mean ± standard deviation for continuous variables. Ranked variables such as RSS and VAS were presented as median and interquartile ranges. Statistical analyses were performed using SPSS for windows version 20.0 (IBM, United States). The normal distribution of the data was tested by Kolmogorov–Smirnov test. Categorical variables between these two groups were analyzed by the χ^2^ test; continuous variables between these two groups were analyzed by the independent *t*-tests; HR, MBP, RR, and SpO_2_ of these two groups at different time points were analyzed by repeated-measures ANOVA followed by Bonferroni *post-hoc* test. The differences of RSS and VAS at different time points were analyzed by Friedman’s test in each group. Mann–Whitney U test was used to analyze the differences of RSS and VAS between these two groups at different time points. For all observations, *α* < 0.05 was accepted as statistically significant.

## Results

**FIGURE 1:**
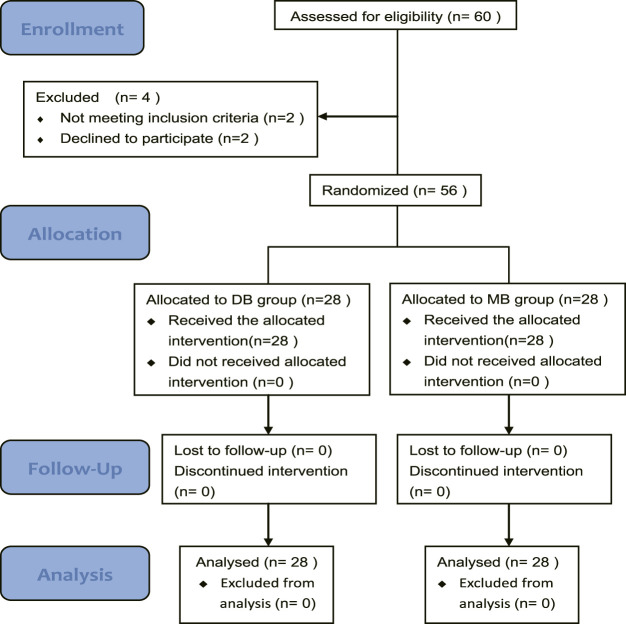
CONSORT flow diagram of the trial. Abbreviation: DB, dexmedetomidine-butorphanol; MB, midazolam-butorphanol.

A total of 60 burn patients needing dressing changes were assessed for eligibility, and four patients were excluded. Finally, 56 patients were enrolled in this study. 28 patients were allocated to group DB and 28 patients were allocated to SB (Figure 1). Patients enrolled in this study were injured by flame, hot water, or chemical, their burn wounds varied from second degree to third degree. There were no significant differences among these groups regarding age, sex, weight, ASA class, etiology, and procedure time (Table 1).

**TABLE 1:** Demographic data of the trial.

	Group DB (*n* = 28)	Group MB (*n* = 28)	Values of *p*
Age (years)	42.25 ± 10.02	42.17 ± 10.65	0.979
Sex (M/F)	19/9	17/11	0.577
ASA(n)			
Ⅰ	13	12	0.788
Ⅱ	15	16	
Weight (kg)	70.00 ± 5.22	69.17 ± 5.27	0.561
TBSA (%)	19.14 ± 5.01	20.60 ± 5.20	0.288
Etiology			
Flame	12	13	0.784
Scald	10	11	
Chemical	6	4	
Procedure time (min)	31.89 ± 4.53	31.17 ± 4.22	0.544

Data are displayed in mean ± SD, or *n* (%). Abbreviation: DB, dexmedetomidine-butorphanol; MB, midazolam-butorphanol; ASA, American society of anaesthesiologists physical status; TBSA, total body surface area.

In the DB group, a significant difference in RSS was observed from baseline to the end of the procedure (χ^2^ = 79.528, *p* < 0.05). In the MB group, the difference of RSS was also significant (χ^2^ = 78.494, *p* < 0.05). The baseline RSS of these two groups were comparable (*p* = 0.284). Sedation scales increased after drug administration. Differences in RSS between these two study groups were not significant at other time points (Table 2, *p* > 0.05).

**TABLE 2:** Ramsay sedation scales of the two study groups.

	Group DB (*n* = 28)	Group MB (*n* = 28)	Values of *p*
Baseline	1 [1, 2]	1.5 [1, 2]	0.284
5 min	3 [3, 4]	3 [3, 4]	0.790
10 min	4 [3, 4]	3.5 [3, 4]	0.178
15 min	4 [3, 4]	4 [3, 4]	0.571
20 min	4 [3, 4]	4 [3, 4]	0.412
25 min	3 [3, 4]	4 [3, 4]	0.108

Data are displayed in median and interquartile ranges. Abbreviation: DB, dexmedetomidine-butorphanol; MB, midazolam-butorphanol.

A significant difference of VAS was observed from baseline to the end of the procedure in the DB group (χ^2^ = 123.167, *p* < 0.05). The difference of VAS was also significant in the MB group (χ^2^ = 120.563, *p* < 0.05). The baseline VAS of these groups was comparable (*p* = 0.382). Pain decreased after drug administration. At other time points, differences in RSS between these two study groups were not significant (Table 3, *p* > 0.05).

**TABLE 3:** Visual analogue scales of the two study groups.

	Group DB (*n* = 28)	Group MB (*n* = 28)	Values of *p*
Baseline	1 [0.25, 1]	1 [0, 1]	0.382
5 min	1 [0.25, 1]	1 [0, 1]	0.656
10 min	5 [4, 5]	4.5 [4, 5]	0.971
15 min	4 [3, 5]	4 [4, 5]	0.197
20 min	4 [4, 5]	4 [3.25, 5]	0.416
25 min	4 [3, 4]	3 [3, 4]	0.736

Data are displayed in median and interquartile ranges. Abbreviation: DB, dexmedetomidine-butorphanol; MB, midazolam-butorphanol.

**FIGURE 2:**
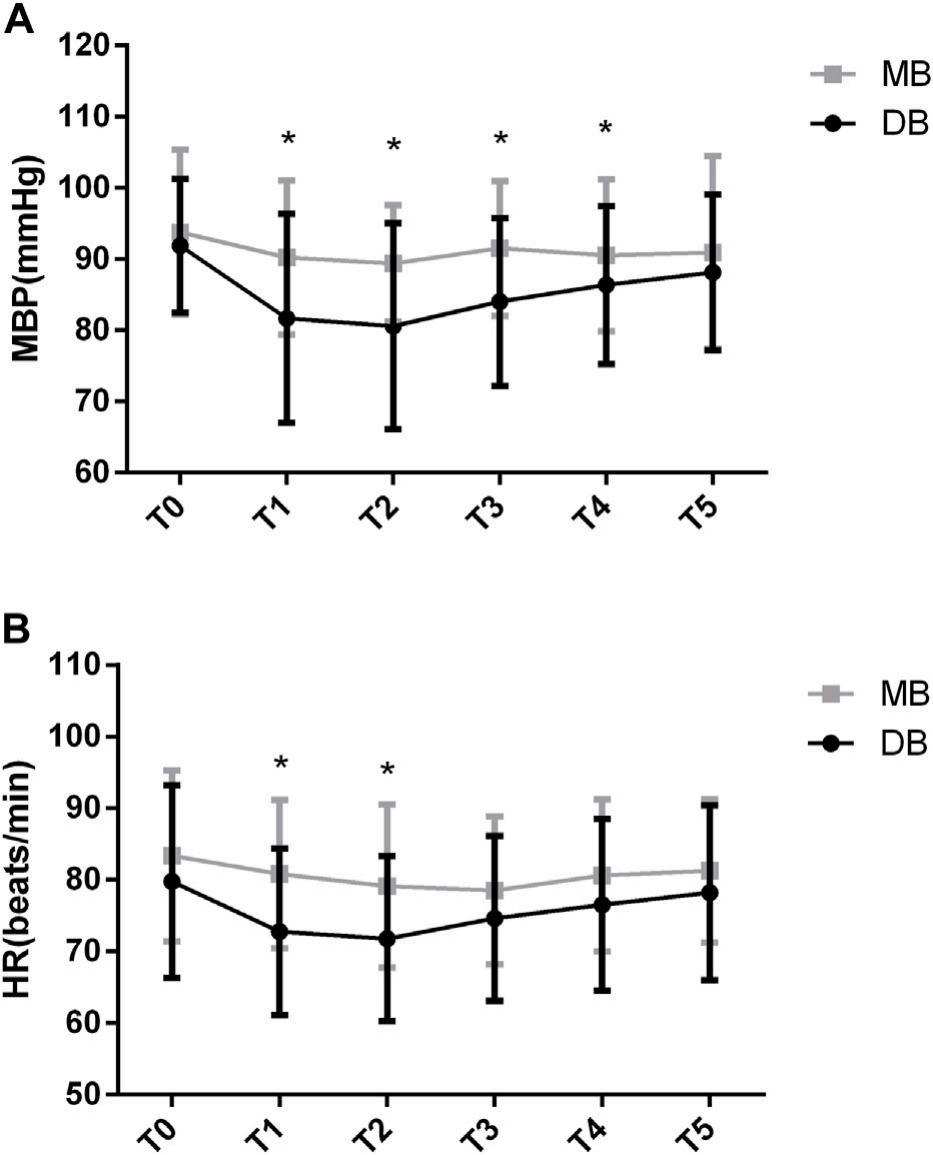
MBP and HR values of the two groups. Abbreviation: DB, dexmedetomidine- butorphanol; MB, midazolam-butorphanol; T0: baseline, T1: beginning, T2: 5 min, T3: 10 min, T4: 15 min, T5: 20 min. *p < 0.05 compared with the MB group.

The difference of MBP at different time points was significant (F = 25.302, *p* < 0.05). The difference in MBP between groups was also significant (F = 46.116, *p* < 0.05). There was significant group-by-time interaction on MBP (F = 2.762, *p* = 0.021). According to the *Post-hoc* test, there were no significant differences regarding the baseline MBP between these two groups (*p* = 0.321). The MBP decreased significantly in the DB group at the beginning (81.6 ± 14.6 vs. 90.2 ± 10.8, *p* < 0.05), at 5 min (80.5 ± 14.5 vs. 89.3 ± 8.1, *p* < 0.05), at 10 min (84 ± 11.7 vs. 91.5 ± 9.4, *p* < 0.05), and at 15 min (86.3 ± 11.1 vs. 90.5 ± 10.7, *p* < 0.05) of the procedure. At 20 min of the procedure, the differences were not significant (Figure 2A, *p* > 0.05).

The between-group factor on HR was significant (F = 5.419, *p* < 0.05). The group-by-time interaction was significant in HR (F = 2.336, *p* = 0.045). *Post-hoc* tests show that there were no significant differences regarding the baseline HR between these two groups (*p* = 0.173). The HR decreased significantly in the DB group at the beginning (72.7 ± 11.6 vs. 80.8 ± 10.3, *p* < 0.05) and 5 min of the procedure (71.7 ± 11.4 vs. 79.1 ± 11.3, *p* < 0.05). At other time points of the procedure, the differences were not significant (Figure 2B, *p* > 0.05).

**FIGURE 3:**
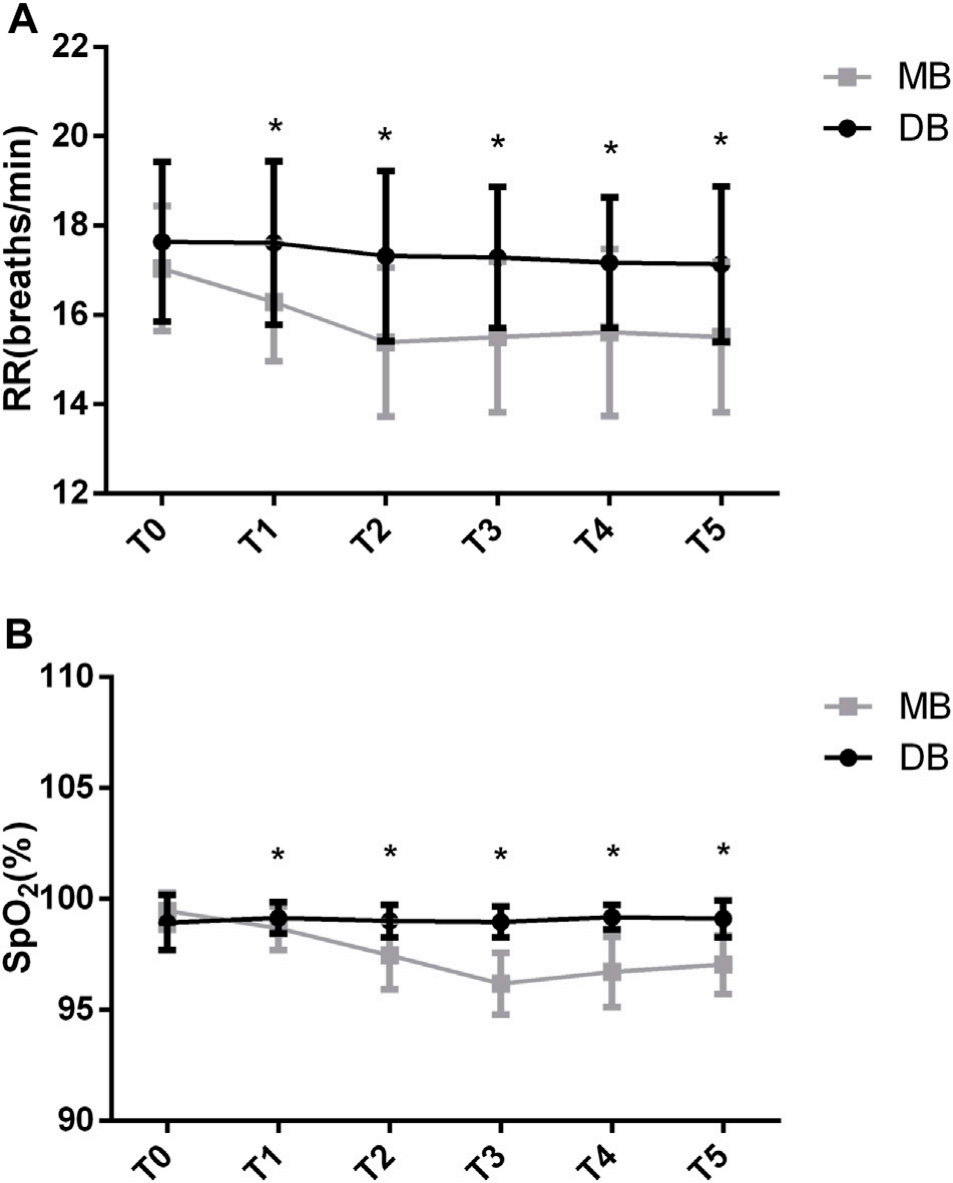
RR and SPO_2_ values of the two groups. Abbreviation: DB, dexmedetomidine- butorphanol; MB, midazolam-butorphanol; T0: baseline, T1: beginning, T2: 5 min, T3: 10 min, T4: 15 min, T5: 20 min. *p < 0.05 compared with the DB group.

The between-group factor on RR was significant (F = 16.521, *p* < 0.05). The group-by-time interaction on RR was significant (F = 3.609, *p* < 0.05). There was a significant drop in RR in the MB group that was significantly different than in the DB group (F = 8.177, *p* < 0.05). According to the *Post-hoc* test, the baseline RR was not significantly different between these two groups (*p* = 0.143). The RR decreased significantly in the MB group at the beginning (16.2 ± 1.3 vs. 17.6 ± 1.8, *p* < 0.05), at 5 min (15.3 ± 1.6 vs. 17.3 ± 1.9, *p* < 0.05), at 10 min (15.5 ± 1.6 vs. 17.2 ± 1.5, *p* < 0.05), at 15 min (15.6 ± 1.8 vs. 17.1 ± 1.4, *p* < 0.05) and at 20 min (15.5 ± 1.6 vs. 17.1 ± 1.7, *p* < 0.05) of the procedure (Figure 3A).

The between-group factor on SpO_2_ was significant (F = 63.380, *p* < 0.05). There was significant group-by-time interaction on SpO_2_ (F = 25.058, *p* < 0.05). There was a significant drop in SpO_2_ in the MB group that was significantly different than in the DB group (F = 26.528, *p* < 0.05). *Post-hoc* test shows that the baseline SpO_2_ of these two groups was not significantly different (*p* = 0.053). The SpO_2_ decreased significantly in the MB group at the beginning (98.6 ± 0.9 vs. 99.1 ± 0.7, *p* < 0.05), at 5 min (97.4 ± 1.5 vs. 99 ± 0.7, *p* < 0.05), at 10 min (96.1 ± 1.3 vs. 98.9 ± 0.6, *p* < 0.05), at 15 min (96.7 ± 1.5 vs. 99.1 ± 0.5, *p* < 0.05) and at 20 min (97 ± 1.3 vs. 99.1 ± 0.8, *p* < 0.05) of the procedure (Figure 3B).

**FIGURE 4:**
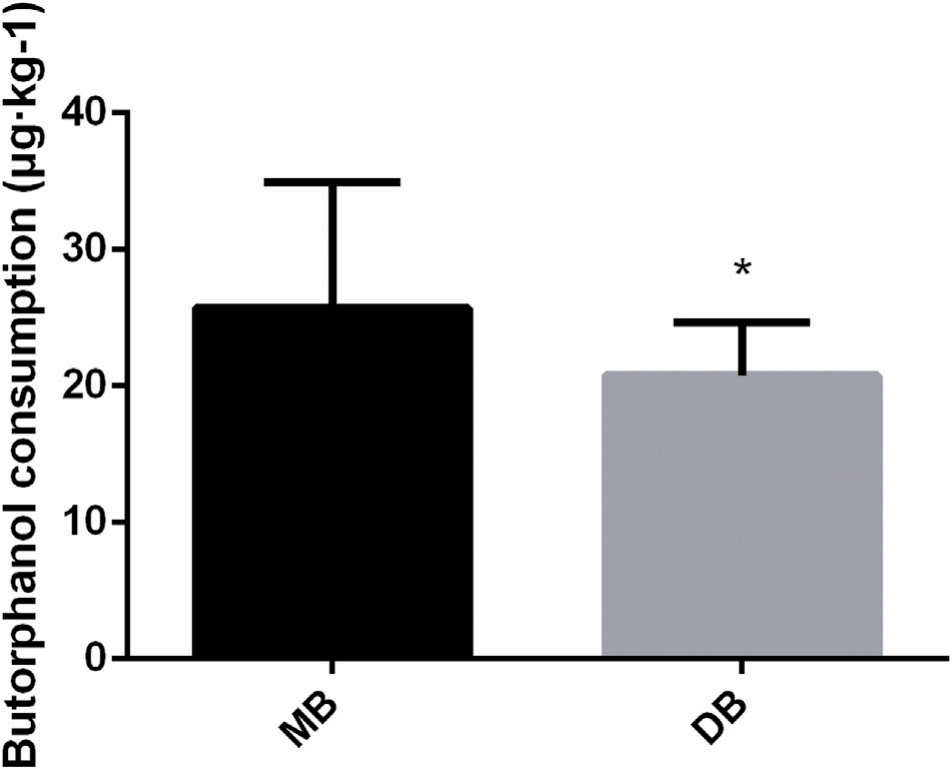
Butorphanol consumption of these two groups. Abbreviation: DB, dexmedetomidine- butorphanol; MB, midazolam-butorphanol. *p < 0.05 compared with the MB group.

The consumption of butorphanol was lower in the DB group than in the MB group (Figure 4, *p* < 0.05). Adverse effects were presented in Table 4. Three patients in the DB group and five patients in the MB group reported nausea and vomiting (*p* > 0.05). Respiratory depression occurred in four patients of the MB group (*p* < 0.05). Hypotension happened more frequently in the DB group (*p* = 0.01).

**TABLE 4:** Side effects of the two study groups.

	Group DB (*n* = 28)	Group MB (*n* = 28)	Values of *p*
Nausea and vomiting	3	5	0.445
Hypotension	6	0	0.01*
Bradycardia	0	0	
Dizziness	0	0	
Respiratory depression	0	4	0.038*
Desaturation	0	0	

Abbreviation: DB, dexmedetomidine-butorphanol; MB, midazolam-butorphanol. *p < 0.05 compared with Group DB.

## Discussion

From this study, we demonstrated that when combined with butorphanol, dexmedetomidine was comparable with midazolam in providing enough sedation and analgesia during burn dressing changes. The benefit of dexmedetomidine lies in its opioid-sparing effect.

The increase in catecholamine levels after burn injury increases resting energy expenditures and myocardial oxygen consumption (Williams and Herndon, 2017). Wang et al. (2019) reported that dexmedetomidine infusion attenuated perioperative stress by inhibiting the secretion of epinephrine and norepinephrine. From the above findings, we speculated that dexmedetomidine can alleviate the hypermetabolic response after burn injury. Dexmedetomidine has a mechanism of action similar to clonidine, but its affinity for the α2 receptor is eight times greater (Canpolat et al., 2012). Hypotension and bradycardia caused by dexmedetomidine were obvious in the previous study (Fonseca et al., 2021); these effects are also dose-dependent. Poorzamany Nejat Kermany et al. (2016) reported that dexmedetomidine provides sufficient analgesia at a bolus dose of 0.5 μg kg^−1^, and its hemodynamic side effects can be alleviated in this approach. Lee et al. (2015) found that a single dose of dexmedetomidine given before induction of anesthesia still works well. Dexmedetomidine has a half-life of 2 h, and its peak effect can be reached within 10 min. Because most of our procedures finished in less than 30 min, dexmedetomidine was given in a bolus dose of 0.5 μg kg^−1^ in the present study, and the maintenance infusion was not needed. From our results, hypotension and bradycardia can still be observed but rarely need medical intervention. We demonstrated that a low dose of dexmedetomidine in the combination of butorphanol can be safely used during burn dressing change, and this combination usage can alleviate the hypermetabolic response after burn injury.

Dexmedetomidine was reported by Zhang et al. (2022) for its low respiratory depression. In our study, this phenomenon was also observed. It can be explained that dexmedetomidine acts primarily on the locus ceruleus of the pons (Nishizawa et al., 2015). The arousable sedation induced by dexmedetomidine is preferred during burn dressing changes. Since most of the burn patients are non-ventilated, safety is more guaranteed by this property. Inconsistent with the previous study (Cho et al. 2014), respiratory depression was more obvious in the MB group. It can be explained that midazolam produces its effects through the Gamma-aminobutyric acid receptors in the brain (Pasin et al., 2015). We, therefore, recommend the combined use of midazolam with butorphanol in critically ill burn patients undergoing mechanical ventilation.

Butorphanol is a synthetic opioid with strong κ-receptor agonist activity. It has both spinal analgesia and sedative functions. Common adverse effects of butorphanol are nausea and vomiting, which are dose-dependent. To reduce the side effects of opioids, the use of opioids in combination with non-opioids is recommended (Lavrentieva et al., 2017). The opioid-sparing effect of dexmedetomidine has been reported by previous studies (Jones et al., 2014). Contrary to dexmedetomidine, during burn dressing changes, the opioid-sparing effect of midazolam was not obvious (Bidwell et al., 2013). There is a concern of over sedation caused by the combination use of dexmedetomidine and butorphanol. In our study, oversedation was not observed. This may partly be attributed to the low dose of dexmedetomidine we administrated. However, care should be taken when a higher dose of dexmedetomidine was administrated.

In the study of Gencer and Sezen (2021), 1 μg kg^−1^ dexmedetomidine or 0.03 mg/kg midazolam was administered before the dressing change procedure. Hemodynamic depression was observed in the dexmedetomidine group and respiratory depression was observed in the midazolam group. In our study, dexmedetomidine was administered at a lower dose, so the hemodynamic depression was alleviated. Different from their study, we performed no debridement in the procedure so that the pain was relieved. Fagin et al. (2012) reported that dexmedetomidine provides more effective sedation for pediatric burn patients when compared with midazolam. They found that patients administered with dexmedetomidine need less mechanical ventilation than with midazolam. We have known that like midazolam, dexmedetomidine has moderately slow pharmacokinetics (Barends et al., 2017). Elkalla and El Mourad (2020) reported that dexmedetomidine was associated with a longer recovery period when compared with a combination of midazolam and fentanyl. The difference in recovery time was not observed between these two groups in our study. We assume that this was attributed to the bolus dose of dexmedetomidine we administered.

Of note, there were limitations in our study. First, we only compared the differences between these two regimens in adult burn patients; their differences between pediatric burn patients were not revealed. Second, a low dose of dexmedetomidine was compared with midazolam in this study. A high dose of dexmedetomidine versus midazolam when combined with butorphanol needs further research. Third, tolerances of butorphanol were not compared between these two groups due to the design of this study. Further study should be carried out to clarify this difference.

## Conclusion

We found that when combined with butorphanol, both dexmedetomidine and midazolam can provide enough sedation and analgesia for burn dressing changes. Dexmedetomidine has more hemodynamic disturbance than midazolam, but it has less respiratory depression than midazolam. The advantage of dexmedetomidine lies in its opioid-sparing effect.

## Data Availability

The raw data supporting the conclusion of this article will be made available by the authors, without undue reservation.

## Abbreviations

CONSORT, consolidated standards of reporting trials; ASA, American society of anesthesiologists; DB, dexmedetomidine-butorphanol; HR, heart rate; MB, midazolam-butorphanol; MBP, mean blood pressure; RR, respiratory rate; RSS, Ramsay sedation scale; SpO_2_, pulse blood oxygen saturation; TBSA, total body surface area; VAS, visual analogue scale.
